# Evaluation of Digitalisation in Healthcare and the Quantification of the “Unmeasurable”

**DOI:** 10.1007/s11606-023-08405-y

**Published:** 2023-09-15

**Authors:** Kathrin Cresswell, Stuart Anderson, Catherine Montgomery, Christopher J. Weir, Marek Atter, Robin Williams

**Affiliations:** 1 Usher Institute, The University of Edinburgh, Edinburgh, UK; 2 School of Informatics, The University of Edinburgh, Edinburgh, UK; 3 Institute for the Study of Science, Technology and Innovation, The University of Edinburgh, Edinburgh, UK; 4 Edinburgh Clinical Trials Unit, Usher Institute, The University of Edinburgh, Edinburgh, UK

## Abstract

Evaluating healthcare digitalisation, where technology implementation and adoption transforms existing socio-organisational processes, presents various challenges for outcome assessments. Populations are diverse, interventions are complex and evolving over time, meaningful comparisons are difficult as outcomes vary between settings, and outcomes take a long time to materialise and stabilise. Digitalisation may also have unanticipated impacts. We here discuss the limitations of evaluating the digitalisation of healthcare, and describe how qualitative and quantitative approaches can complement each other to facilitate investment and implementation decisions. In doing so, we argue how existing approaches have focused on measuring what is easily measurable and elevating poorly chosen values to inform investment decisions. Limited attention has been paid to understanding processes that are not easily measured even though these can have significant implications for contextual transferability, sustainability and scale-up of interventions. We use what is commonly known as the McNamara Fallacy to structure our discussions. We conclude with recommendations on how we envisage the development of mixed methods approaches going forward in order to address shortcomings.

## INTRODUCTION

The underlying assumption of digitalisation of healthcare is that it has the potential to improve safety, quality and efficiency (in terms of allocation of resources).^1^ Digitalisation refers to the socio-organisational transformations associated with the implementation and adoption of technology, as opposed to merely automating existing processes.^2^ Expectations drive the frequent assumption that outcomes are measurable changes resulting from an intervention which will in due course deliver impacts. Impacts are the broader effects of an outcome that materialise over time.^3^

Research can measure the outcomes of a digital intervention and this information can help to judge if an intervention has worked. Strategic decision makers, implementers and technology suppliers can then use this information to justify investments and to inform business cases for future initiatives. Healthcare staff are also more likely to adopt a digital intervention if it is evidence-based.^4, 5^

**Figure 1:**
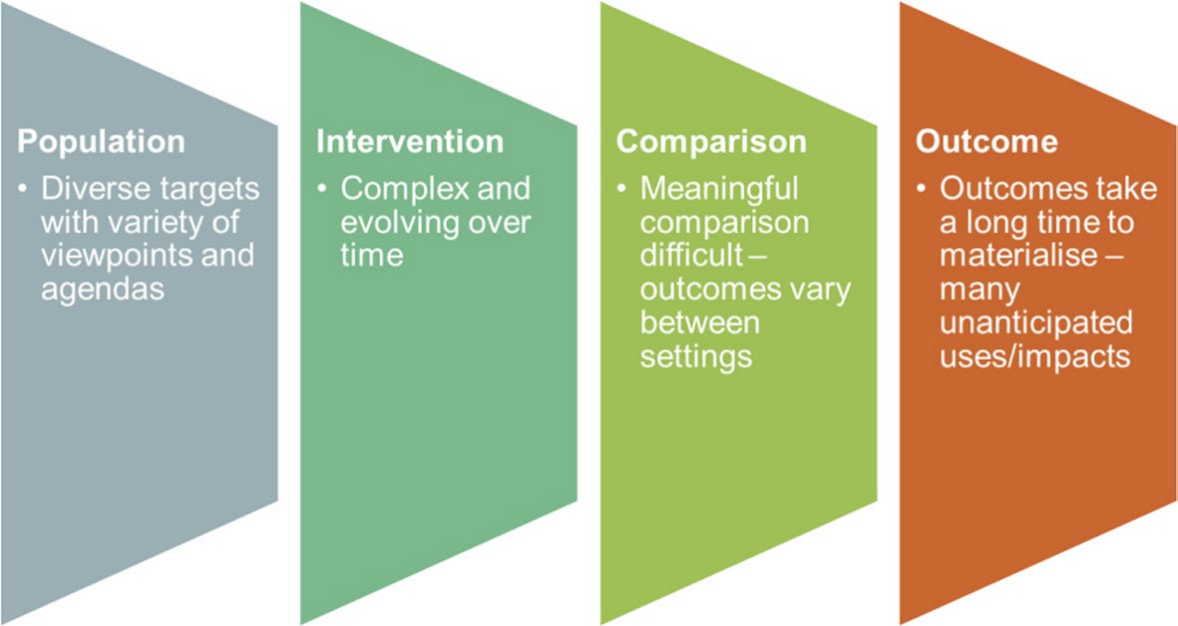
Issues in healthcare digitalisation presenting challenges for quantitative measurement.

However, outcomes and impacts often materialise slowly over extended timeframes, frequently involve health information infrastructures that take a long time to implement and evolve (as opposed to discrete technologies), vary across contexts, and are hard to quantify and difficult to attribute to particular changes. Interventions are also increasingly complex, including various behavioural and educational elements in addition to technological aspects. Finally, although digital initiatives are often seen as delivering specific short-term improvements, they usually also bring unintended outcomes and pave the way for longer-term evolution of services.^6^ For example, risk scoring for a particular event (e.g. readmission for the same condition) can radically change the demand profile on a range of services,^7^ suggesting potentially wide-ranging service redesign. This deep interaction between intervention and context poses significant challenges for quantitative measurement (Fig. 1).^8^

Although existing frameworks acknowledge the importance of mixed methods work in evaluations of complex interventions,^9^ they are rarely implemented effectively. We will here build on existing work advocating the use of mixed methods in evaluating digitalisation initiatives in healthcare settings,^10, 11^ discussing the pitfalls associated with a sole focus on quantitative measurements, and exploring why mixed methods work is still rarely done well.

### The Limitations of Quantification in the Digitalisation of Healthcare

Sociologists, including Daniel Yankelovich, have considered the limitations of quantitative measurements.^12–14^ Yankelovich analysed the behaviours of Robert McNamara, the US Secretary of Defense during the Vietnam war. McNamara attempted to quantify the success of the war through enemy body counts alone. He neglected other factors that could not easily be measured. This is now commonly known as the *McNamara Fallacy*.^14^ Yankelovich proposed four pitfalls relating to limitations in approaches to quantifying outcomes, which we will discuss and apply to healthcare digitalisation in the following sections (Box 1).

**Box 1** Yankelovich’s four pitfalls relating to limitations in approaches to quantifying outcomes

**Table:** 

1. Measuring whatever can be easily measured 2. Adopting particular poorly selected quantitative measures as proxy indicators of values which cannot easily be measured 3. Presuming that what cannot be measured easily is not important 4. Assuming that what cannot be easily measured does not exist	

The first pitfall refers to using easily measurable data to evidence decisions when its relevance to the decision is unclear. In digitalisation projects, this may relate to capturing real-world operational data relating to project management metrics, such as money spent on purchasing and implementing a new system, or the time it takes to deliver a project.^15^ Although useful to monitor progress, these quantitative measurements do not provide a complete assessment of impacts and they fail to account for programmatic aspects that go beyond the confines of a particular change project. For example, the impact of a digital intervention on patient outcomes (e.g. morbidity and mortality) is crucial as an end-point of success, but it is often not measured. This is because patient outcomes often take a long time to materialise (as new technologies embed), are expensive to collect (e.g. evidence of harm from medication errors) and are hard to trace with quantitative means as they may be subjective (e.g. quality of life).

Yankelovich’s second pitfall of quantification refers to giving “*an arbitrary quantitative value*” to outcomes “*which can’t be easily measured*.” The enthusiasm for cost-benefit analysis was originally intended as a way to bring a degree of objectivity to inform political decisions,^16^ and has value where quantitative justifications are needed but other evidence is lacking (e.g. business cases informing investment). However, such quantitative measurements are to a degree artificial, as they only provide partial insights into outcomes. They are proxy measures or, in the clinical trials context, putative surrogate outcomes.^17, 18^ For example, different tools for measuring utility (e.g. quality-adjusted life-years) may produce different results and lead to inconsistencies and errors.^19–22^ Similarly, hospital mortality rates, although commonly used, may not be appropriate to judge a hospital’s performance, as they fail to take into account contextual factors that may influence outcomes (e.g. severity of conditions, patient demographics).^23^

The third pitfall of quantification involves presuming that “*what can’t be measured easily really isn’t important*.” This is where quantitative approaches have real difficulty. For instance, user engagement is clearly important in every digitalisation initiative, and there are many accounts of failed systems that are not used or do not scale in the literature.^24, 25^ However, although crucial for successful implementation, user and patient engagement is very difficult to measure quantitatively.

Yankelovich’s final caution highlights the risk of discounting factors that cannot be effectively quantified. User workarounds are a good example. When faced with a system that disrupts workflows, users tend to either ignore it or, if this is not possible, behave in a way that makes it appear that the system is working. This may include clicking away boxes without reading them in order to get to the next screen. Resulting risks can include threats to patient safety and unreliable data that is problematic for secondary uses.^26, 27^ Rather than ignoring such workarounds, managers need to surface them, in order to mitigate risks to patient safety.^28, 29^

As digital applications become more sophisticated and influence data-driven decisions, there is an exacerbated risk that data that is difficult to measure gets neglected when making decisions.^30^

### The Potential of Mixed Methods in Accounting for the “Unmeasurable”

Mixed methods can be used synergistically to address some of the shortcomings associated with quantification. However, there is currently poor integration of quantitative and qualitative approaches in digital transformation.^31^ Some sociologists have proposed promising directions to study value creation in digital transformation.^31, 32^ This has, for example, been shown to be particularly important in relation to digital exclusion of some groups (who may be unable to fully exploit technologies due to lack of access to devices, reliable internet connections or lack of digital skills), which quantitative approaches have trouble accounting for.^33^

Mixed methods are also critical for understanding the relationship between intervention and the context in which it is delivered. They can help to identify processes that lead to a particular outcome, including why a digital intervention has worked or why it has failed to achieve its anticipated impacts. Qualitative methods can help increase the success of implementation, by identifying the source of potential failures, and aid effective management of change. For example, many potential barriers to effective technology implementation and adoption are socio-organisational and informal in nature and therefore not directly visible or measurable. These may include discovering through interviews or observations how users employ workarounds to compensate for lack of usability of systems.

Table 1 illustrates some examples of mixed methods studies in digitalisation and the contribution of different components.

**Table 1:** Examples of Mixed Methods Studies

Reference	Study aim	Quantitative component	Qualitative component
Watkinson F, Dharmayat KI, Mastellos N. A mixed-method service evaluation of health information exchange in England: technology acceptance and barriers and facilitators to adoption. BMC health services research. 2021 Dec;21(1):1–3	To assess the extent to which users embraced Health Information Exchange (HIE) and to investigate the factors that either hindered or supported its broader adoption	Questionnaire to assess technology acceptance	Semi-structured interviews to explore barriers and facilitators to adoption
Powell KR, Deroche CB, Alexander GL. Health data sharing in US nursing homes: a mixed methods study. Journal of the American Medical Directors Association. 2021 May 1;22(5):1052–9	To explore nursing homes’ capability for data sharing and leaders’ perceptions surrounding data sharing	Secondary analysis of survey data	Semi-structured interviews to explore perceived challenges and benefits of data sharing
Murphy DR, Satterly T, Giardina TD, Sittig DF, Singh H. Practicing clinicians’ recommendations to reduce burden from the electronic health record inbox: a mixed-methods study. Journal of general internal medicine. 2019 Sep 15;34:1825–32	To identify electronic health record inbox design strategies	Secondary analysis of survey data to extract design suggestions	Interviews to understand strategies and improve efficiency
Clarke MA, Fruhling AL, Sitorius M, Windle TA, Bernard TL, Windle JR. Impact of age on patients’ communication and technology preferences in the era of meaningful use: mixed methods study. Journal of medical Internet research. 2020 Jun 1;22(6):e13470	To identify patient preferences in relation to the use of information and communication technology	Instruments measuring health literacy, confidence using technology, and patient activation	Semi-structured interviews to explore current care processes
Weijers M, Boumans N, van der Zwet J, Feron F, Bastiaenen C. A feasibility Randomised Controlled Trial as a first step towards evaluating the effectiveness of a digital health dashboard in preventive child health care: a mixed methods approach. Pilot and Feasibility Studies. 2023 Feb 15;9(1):25	To evaluate the feasibility of exploring the effectiveness of a digital health dashboard in preventive child health care	Feasibility randomised controlled trial	Semi-structured interviews to explore quantitative findings

Digitalisation encounters unexplained variation across contexts, which are not adequately addressed in many traditional quantitative studies. For example, research has shown that high-level leadership can help to ensure that a digital transformation initiative is successfully implemented and adopted, but this is difficult to measure.^34^ Similarly, recent artificial intelligence–based applications depend heavily on training datasets.^35^ If the local population differs from these training datasets, then the outputs of the application are not reliable in different contexts. Many quantitative studies also treat diverse instances as if they were the same, and thereby ignore the diversity of intermediate (e.g. what is implemented, changes to practices) and ultimate outcomes (e.g. patient experience, health outcomes). Lastly, they cannot account for individual experiences, which can have significant implications for uptake, use and outcomes.^36^

Whilst qualitative methods address some of quantitative methods’ shortcomings, it is important to acknowledge that qualitative methods have their own limitations. For example, they do not use statistical extrapolation as the basis for claimed relevance to decisions. Genuine mixed methods work therefore needs to be synergistic between approaches. Here, qualitative analysis can help to identify appropriate quantitative measures and facilitate the interpretation of quantitative measurements. An example is the Triple C study, which has led to the development of guidelines surrounding the use of case studies to explore complex interventions including digital health initiatives.^37–39^ This study was designed to explore how findings of research from different traditions can be combined. One of the key emerging tensions from this work was how the relationship between context and intervention can be conceptualised.

### Remaining Tensions and Why Measuring the “Unmeasurable” Is Still Difficult

Although valuable,^40^ mixed methods are rarely done well, often either sequentially applying one or the other but failing to explore linkages and interactions, or mechanistically applying one to the other. This may include for example quantifying qualitative findings as is commonly seen in approaches to identify facilitators and barriers in implementation science studies, or aggregating summary outcomes of multiple qualitative studies and then applying statistical analyses. There are underlying tensions between the positivist tradition (associated with quantitative studies) and constructivist/interpretivist traditions (associated with qualitative studies) in how claims are verified and how case study findings may be extrapolated. The positivist tradition is a philosophical and methodological approach that emphasises empirical observation, scientific method and the objective study of the natural and social world, whereas the constructivist/interpretivist tradition highlights the role of individuals’ perspectives, cultural contexts and social interactions in shaping reality.

There are difficulties in promoting scholarship across the two approaches. For example, some mixed methods designs may have been used to support positivist claims and do not sufficiently account for the complexities of the social world including stakeholder relationships and power dynamics.^41, 42^ An example here is the Whole System Demonstrator, a randomised controlled trial investigating the effectiveness of telehealth and telecare. Policy makers interpreted and disseminated the results in a way that fitted their existing rhetoric, stating that it was effective in terms of cost and clinical benefit.^43, 44^ However, telecare was not found to be cost-effective. This may be because studies assumed an unlimited supply of labour, and used outcome measures that may not have represented real impact of systems.

Existing inter-disciplinary silos and tensions also contribute to the lack of high-quality mixed methods studies.^45, 46^ The challenge going forward will be to explore how the two approaches can meaningfully complement each other in the fast-evolving area of digitalisation. Pragmatic approaches offer a common middle ground of exploring and combining the complementary strengths of quantitative and qualitative methods.^47^ In relation to digital health, this may involve combining quantitative impact work with longitudinal formative qualitative process evaluations. Both elements then need to inform overall summative judgements around whether the intervention is worth pursuing. This summative work needs to involve an assessment of effectiveness (the quantitative element) and an assessment of likelihood of transferability, sustainability and scale-up (the qualitative element).

There is now a need to map how these complementary approaches can best be integrated and applied in evaluating digitalisation in healthcare. Mixed methods approaches need to co-produce studies of an intervention with movement back and forth between qualitative and quantitative elements.^48^ This may include using qualitative insights to inform quantitative designs (e.g. through identifying what to measure) and should be characterised by agility, acknowledging that evaluation and intervention shape each other. Key will be appropriate incentives for evaluators and for participants. This may need to involve allowing evaluation flexibility from funders, moving away from the traditional focus on outcome assessment to more balanced mixed methods and (where relevant) qualitative approaches. In doing so, researchers may need to distinguish between the focus of investigation, and their operationalisation of measuring outcomes associated with that focus. This will help to distinguish the latent concept and the measurement, helping to reveal limitations of the measure, particularly when this is quantitative. Here, qualitative methods can help to characterise the focus of investigation, and inform outcome assessment by identifying which outcome measures most adequately capture the phenomenon of interest.

Despite a degree of recognition of the importance of mixed methods amongst researchers, quantitative designs often take immediate priority in digitalisation initiatives, as they seem to provide the information needed to justify investment decisions.^49^ Scaling and sustainability considerations are unfortunately often only considered when problems are encountered. Here, quantitative methods may treat implementation contexts as comparable, but fail to take into account factors that may prove to be key barriers to scaling and sustainability. This is exemplified by the many current investments in artificial intelligence, and the relatively poor characterisation of implementation and adoption contexts in this area.^50^ As we move into the development of increasingly complex heterogeneous data sets, we may see a shift from coarse-grained, analytically tractable, quantitative models to fine-grained simulation models that are based on mixed methods insights. Quantitative methods and results are often perceived by strategic decision makers as more convincing, objective and valid than qualitative methods and results. In addition, the assumptions underpinning quantitative arguments are often omitted and can be used to mislead. To address this barrier, and for qualitative and mixed methods work to be more widely adopted, there is a need to promote awareness of and training in qualitative methods and their validation amongst decision makers.

## CONCLUSIONS

We hope that this publication can inform ongoing research designs and help to inform strategic decisions surrounding investments in digitalisation of healthcare and beyond. Decision-makers need to build business models to justify investments in a context of incomplete information and are forced to extrapolate from poorly chosen available proxy measures. They tend to fail to ask questions around contextual and processual factors that affect scaling and sustainability, the kinds of questions that can be answered with qualitative designs.

Therefore, there is now a need to build new approaches for designing and appraising digitalisation in healthcare. This may involve developing pragmatic approaches to address this issue and raise the profile of mixed methods and the risk associated with simplistic resort to quantification amongst the policy community. Such efforts will help to ensure evidence-based policymaking and promote the effective positive transformation of healthcare through digitalisation.

## References

[1] **Chaudhry B, Wang J, Wu S, Maglione M, Mojica W, Roth E, Morton SC, Shekelle PG**. Systematic review: impact of health information technology on quality, efficiency, and costs of medical care. Ann Intern Med. 2006;144(10):742-52 10.7326/0003-4819-144-10-200605160-00125 16702590

[2] Digitalization and Digitization. Available from: https://culturedigitally.org/2014/09/digitalization-and-digitization/. Last accessed 10/08/2023.

[3] What is the difference between an impact and an outcome? Impact is the longer term effect of an outcome. Available from: https://blogs.lse.ac.uk/impactofsocialsciences/2014/10/27/impact-vs-outcome-harding/. last accessed: 10/05/2023.

[4] **Cresswell K, Sheikh A**. Organizational issues in the implementation and adoption of health information technology innovations: an interpretative review. Int J Med Inform. 2013;82(5):e73-86. 10.1016/j.ijmedinf.2012.10.007 23146626

[5] **Black AD, Car J, Pagliari C, Anandan C, Cresswell K, Bokun T, McKinstry B, Procter R, Majeed A, Sheikh A**. The impact of eHealth on the quality and safety of health care: a systematic overview. PLoS Med. 2011;8(1):e1000387. 10.1371/journal.pmed.1000387 PMC3022523 21267058

[6] **Lyytinen K, Sørensen C, Tilson D**. Generativity in digital infrastructures: a research note. In The Routledge companion to management information systems 2017 Aug 15 (pp. 253–275). Routledge.

[7] **Alam N, Hobbelink EL, van Tienhoven AJ, van de Ven PM, Jansma EP, Nanayakkara PW**. The impact of the use of the Early Warning Score (EWS) on patient outcomes: a systematic review. Resuscitation. 2014;85(5):587-94. 10.1016/j.resuscitation.2014.01.013 24467882

[8] How to clarify a clinical question. Available from: https://bestpractice.bmj.com/info/toolkit/learn-ebm/how-to-clarify-a-clinical-question/. last accessed: 10/05/2023.

[9] **Skivington K, Matthews L, Simpson SA, Craig P, Baird J, Blazeby JM, Boyd KA, Craig N, French DP, McIntosh E, Petticrew M**. A new framework for developing and evaluating complex interventions: update of Medical Research Council guidance. BMJ 2021;374:n2061. 10.1136/bmj.n2061 PMC8482308 34593508

[10] **Sockolow P, Dowding D, Randell R, Favela J**. Using mixed methods in health information technology evaluation. Stud Health Technol Inform. 2016;225:83-7. 27332167

[11] **Scott PJ**. Mixed methods: a paradigm for holistic evaluation of health IT. Stud Health Technol Inform. 2016;222:102-13. 27198096

[12] **Espeland WN, Stevens ML**. A sociology of quantification. European Journal of Sociology/Archives européennes de sociologie. 2008;49(3):401-36.

[13] **Porter TM**. Trust in numbers: the pursuit of objectivity in science and public life. Princeton, NJ: Princeton University Press; 1995. 10.1177/030631299029004007 11623934

[14] **Yankelovich D**. Corporate priorities: a continuing study of the new demands on business. Stanford, CT: Yankelovich Inc; 1972.

[15] **Palfreyman J, Morton J**. The benefits of agile digital transformation to innovation processes. Journal of Strategic Contracting and Negotiation. 2022;6(1):26-36.

[16] **Porter TM, Haggerty KD**. Trust in numbers: the pursuit of objectivity in science & public life. Can J Sociol. 1997;22(2):279.

[17] **Taylor RS, Elston J**. The use of surrogate outcomes in model-based cost-effectiveness analyses: a survey of UK health technology assessment reports. Health Technol Assess. 2009;13(8):1-50. 10.3310/hta13080 19203465

[18] **Ciani O, Buyse M, Drummond M, Rasi G, Saad ED, Taylor RS**. Time to review the role of surrogate endpoints in health policy: state of the art and the way forward. Value Health. 2017;20:487-495. 10.1016/j.jval.2016.10.011 28292495

[19] **Duru G, Auray JP, Béresniak A, Lamure M, Paine A, Nicoloyannis N**. Limitations of the methods used for calculating quality-adjusted life-year values. Pharmacoeconomics. 2002;20:463-73. 10.2165/00019053-200220070-00004 12093302

[20] **Kaplan R**. Utility assessment for estimating quality-adjusted life years. In F. Sloan (Ed.), Valuing Health Care: Costs, Benefits, and Effectiveness of Pharmaceuticals and Other Medical Technologies (pp. 31-60). Cambridge: Cambridge University Press; 1995. 10.1017/CBO9780511625817.003.

[21] **Drummond M, Brixner D, Gold M, Kind P, McGuire A, Nord E**, Consensus Development Group. Toward a consensus on the QALY. Value in Health. 2009;12:S31–5. 10.1111/j.1524-4733.2009.00522.x 19250129

[22] **Carlson JJ, Brouwer ED, Kim E, Wright P, McQueen RB**. Alternative approaches to quality-adjusted life-year estimation within standard cost-effectiveness models: literature review, feasibility assessment, and impact evaluation. Value in Health. 2020;23(12):1523-33. 10.1016/j.jval.2020.08.2092 33248507

[23] **O’Mahony S**. Medicine and the McNamara Fallacy. J R Coll Physicians Edinburgh. 2017;47(3):281-7. 10.4997/JRCPE.2017.315 29465108

[24] **Cho Y, Kim M, Choi M**. Factors associated with nurses’ user resistance to change of electronic health record systems. BMC Medical Inform Decis Mak. 2021;21(1):1-2. 10.1186/s12911-021-01581-z PMC8286589 34273990

[25] **Clawson J, Pater JA, Miller AD, Mynatt ED, Mamykina L**. No longer wearing: investigating the abandonment of personal health-tracking technologies on craigslist. In: Proceedings of the 2015 ACM international joint conference on pervasive and ubiquitous computing. 2015. p. 647-658.

[26] **Kilkenny MF, Robinson KM**. Data quality: “garbage in–garbage out”. Health Inf Manag J. 2018;47(3):103-5. 10.1177/1833358318774357 29719995

[27] **Koppel R, Wetterneck T, Telles JL, Karsh BT**. Workarounds to barcode medication administration systems: their occurrences, causes, and threats to patient safety. J Am Med Inform Assoc. 2008;15(4):408-23. 10.1197/jamia.M2616 PMC2442264 18436903

[28] **Cresswell KM, Mozaffar H, Lee L, Williams R, Sheikh A**. Workarounds to hospital electronic prescribing systems: a qualitative study in English hospitals. BMJ Qual Saf. 2017;26(7):542-51. 10.1136/bmjqs-2015-005149 27129493

[29] **Feldman MS**. Organizational routines as a source of continuous change. Organ Sci. 2000;11(6):611-29.

[30] **Hassani H, Huang X, MacFeely S**. Impactful digital twin in the healthcare revolution. Big Data Cogn Comput. 2022;6(3):83.

[31] **Marent B, Henwood F**. Digital health: a sociomaterial approach. Sociol Health Illn. 2023;45(1):37– 53. 10.1111/1467-9566.13538. 10.1111/1467-9566.13538 PMC10088008 36031756

[32] **Datta Burton S, Kieslich K, Paul KT et al**. Rethinking value construction in biomedicine and healthcare. BioSocieties. 2022;17:391–414. 10.1057/s41292-020-00220-6.

[33] **Prainsack B**. Logged out: ownership, exclusion and public value in the digital data and information commons. Big Data Soc. 2019;6(1):2053951719829773.

[34] **Greenhalgh T, Stramer K, Bratan T, Byrne E, Mohammad Y, Russell J**. Introduction of shared electronic records: multi-site case study using diffusion of innovation theory. Bmj. 2008;337:a1786. 10.1136/bmj.a1786 PMC3269664 18948344

[35] **Yu KH, Beam AL, Kohane IS**. Artificial intelligence in healthcare. Nat Biomed Eng. 2018;2(10):719-31. 10.1038/s41551-018-0305-z 31015651

[36] **Sicotte C, Paré G**. Success in health information exchange projects: solving the implementation puzzle. Soc Sci Med. 2010;70(8):1159-65. 10.1016/j.socscimed.2009.11.041 20137847

[37] **Green J, Hanckel B, Petticrew M, et al**. Case study research and causal inference. BMC Med Res Methodol. 2022;22, 307. 10.1186/s12874-022-01790-8. 10.1186/s12874-022-01790-8 PMC9714179 36456923

[38] **Paparini S, Green J, Papoutsi C, et al**. Case study research for better evaluations of complex interventions: rationale and challenges. BMC Med. 2020;18:301. 10.1186/s12916-020-01777-6. 10.1186/s12916-020-01777-6 PMC7652677 33167974

[39] **Paparini S, Papoutsi C, Murdoch J, et al**. Evaluating complex interventions in context: systematic, meta-narrative review of case study approaches. BMC Med Res Methodol; 2021;21:225. 10.1186/s12874-021-01418-3 PMC8543916 34689742

[40] **Scott P**. Mixed methods: a paradigm for holistic evaluation of health IT. In: Evidence-Based Health Informatics: Promoting Safety and Efficiency Through Scientific Methods and Ethical Policy. Germany: IOS Press; 2016. 27198096

[41] **Greenhalgh T, Russell J**. Why do evaluations of eHealth programs fail? An alternative set of guiding principles. PLoS Med. 2010;7(11):e1000360. 10.1371/journal.pmed.1000360 PMC2970573 21072245

[42] **Giddings LS**. Mixed methods research: positivism dressed in drag? J Res Nurs. 2006;11(3):195–203.

[43] Making a reality of telehealth: lessons from the Whole System Demonstrator programme. Available from: https://www.kingsfund.org.uk/blog/2011/11/making-reality-telehealth-lessons-whole-system-demonstrator-programme. last accessed: 10/05/2023.

[44] **Greenhalgh T**. Whole System Demonstrator trial: policy, politics, and publication ethics. BMJ. 2012;345:e5280. 10.1136/bmj.e5280 22868961

[45] **Mykhalovskiy E, Frohlich KL, Poland B, Di Ruggiero E, Rock MJ, Comer L**. Critical social science with public health: agonism, critique and engagement. Crit Public Health. 2019;29(5):522-33.

[46] **Arghode V**. Qualitative and Quantitative Research: Paradigmatic Differences. Global Education Journal 2012(4):155–63.

[47] **Onwuegbuzie AJ, Leech NL**. On becoming a pragmatic researcher: the importance of combining quantitative and qualitative research methodologies. Int J Soc Res Methodol. 2005;8(5):375-87.

[48] **Minary L, Alla F, Cambon L, Kivits J, Potvin L**. Addressing complexity in population health intervention research: the context/intervention interface. J Epidemiol Community Health. 2018;72(4):319-23. 10.1136/jech-2017-209921 PMC5868525 29321174

[49] **Tichenor M, Merry SE, Grek S, Bandola-Gill J**. Global public policy in a quantified world: Sustainable Development Goals as epistemic infrastructures. Policy Soc. 2022;41(4):431-44.

[50] **Ellahham S, Ellahham N, Simsekler MC**. Application of artificial intelligence in the health care safety context: opportunities and challenges. Am J Med Qual. 2020;35(4):341-8. 10.1177/1062860619878515 31581790

